# Hemophagocytic lymphohistiocytosis is associated with *Bartonella henselae* infection in a patient with multiple susceptibility genes

**DOI:** 10.1186/s12941-020-00370-2

**Published:** 2020-06-09

**Authors:** Tianjun Yang, Qing Mei, Lei Zhang, Zhendong Chen, Chunyan Zhu, Xiaowei Fang, Shike Geng, Aijun Pan

**Affiliations:** 1 Department of Intensive Care Unit, The First Affiliated Hospital of USTC, Division of Life Science and Medicine, University of Science and Technology of China, 17 Lujiang Road, Hefei, Anhui China; 2 Department of Intensive Care Unit, Affiliated Provincial Hospital of Anhui Medical University, 17 Lujiang Road, Hefei, Anhui China

**Keywords:** Hemophagocytic lymphohistiocytosis, *Bartonella henselae*, Cat-scratch disease, Next-generation sequencing, Genetic defects

## Abstract

**Background:**

Adult-onset hemophagocytic lymphohistiocytosis (HLH) is a rare and life-threatening condition, which is often triggered by certain types of infection, cancer and numerous autoimmune diseases; however, of the numerous infectious triggers associated with HLH, the consequences of *Bartonella henselae* infection have been rarely reported.

**Case presentation:**

A 48-year-old female presented with a 20-day history of intermittent fever accompanied by systemic rash, fatigue, anorexia and weight loss later she developed shock and unconsciousness. Blood tests showed a reduction of leukocyte, anemia and thrombocytopenia, and pathological results of a bone marrow biopsy confirmed hemophagocytic activity. Metagenomic next-generation sequencing (mNGS) analysis of the lymph node detected the presence of *B. henselae*. Whole exome sequencing revealed two gene variants, STXBP2 and IRF5, in this adult patient with secondary HLH. Then, she received minocycline and rifampin combination anti-infective therapy. Intravenous immunoglobulin for 5 days followed by a high dose of methylprednisolone were also administered. The patient was successfully discharged from the intensive care unit and remained in good condition after 2 months of follow-up.

**Conclusions:**

mNGS served crucial roles in obtaining an etiological diagnosis, which suggested that screening for *B. henselae* should be considered in patients with HLH, especially those with a cat at home. In addition, the genetic defects were discovered to not only be present in primary HLH, but also in secondary HLH, even in the elderly.

## Background

Hemophagocytic lymphohistiocytosis (HLH) is a rare, but aggressive and life-threatening syndrome, which is characterized by a hyperinflammatory immune response that initiates systemic inflammation, hypercytokinemia and multi-organ failure [1]. HLH is broadly divided into two distinct groups: Primary and secondary HLH. In adults, secondary HLH is often triggered by certain types of infection, cancer and numerous autoimmune diseases; however, of the numerous infectious triggers associated with HLH, the consequences of *Bartonella henselae (B. henselae)* infection have been rarely reported [2, 3]. *B. henselae* is known to cause cat-scratch disease (CSD), a benign and self-limited condition, which presents in immunocompetent children. However, the condition is hypothesized to worsen if the patients developed hemophagocytic syndrome. Thus, the present case study reported on a case of HLH associated with disseminated *B. henselae* infection in a patient with both STXBP2 and IRF5 gene variants.

## Case presentation

A 48-year-old female with a previous medical history of drug allergies was recently prescribed a traditional Chinese medicine (TCM), namely a Niuhuang Jiedu tablet, for their swollen gums. A painful and itchy purple patch of skin on the lips, genitals and hands occurred within 12 h of taking the drug. Although the patient did not seek medical advice in time, the rash gradually subsided without any medical treatment. On the seventh day after the allergic reaction, the patient presented with a 20-day history of intermittent fever, which was accompanied by another systemic rash, fatigue, anorexia and weight loss. The patient was subsequently admitted to our hospital for further evaluation and management.

On admission, the patient has a temperature of 38.4 °C, a pulse rate of 120 beats/min, a respiratory rate of 22 breaths/min and a blood pressure of 68/36 mmHg; the patient was also unconscious. A physical examination revealed a palpable spleen located 3 cm below the costal margin, and an enlarged, painful and mobile lymph node in the right inguinal region. The chest and heart were normal. Immediately, the emergency team performed anti-shock procedures and further tests. The laboratory evaluation revealed that the patient had a total leukocyte count of 1850 × 10^6^/L, alongside a diagnosis of anemia (hemoglobin, 79 g/L) and thrombocytopenia (platelet count, 2000 × 10^6^/L). Liver function assays were subsequently performed, and the following levels were recorded: (i) Aminotransferase, 69.1 U/L; (ii) aspartate aminotransferase, 182 U/L; (iii) lactate dehydrogenase, 6194 U/L; bilirubin total/direct, 52.8/37.1 μmol/L; and (iv) ferritin, > 2000 ng/mL. The following parameters were also recorded: (i) Cholesterol, 2.51 mmol/L; (ii) triglycerides, 1.81 mmol/L; (iii) prothrombin time, 17.4 s; (iv) partial thromboplastin time, 56.8 s; and (v) fibrinogen, 0.77 g/L. Computed tomography scans revealed scattered, high-density patchy opacities in the bilateral lungs.

**Fig. 1:**
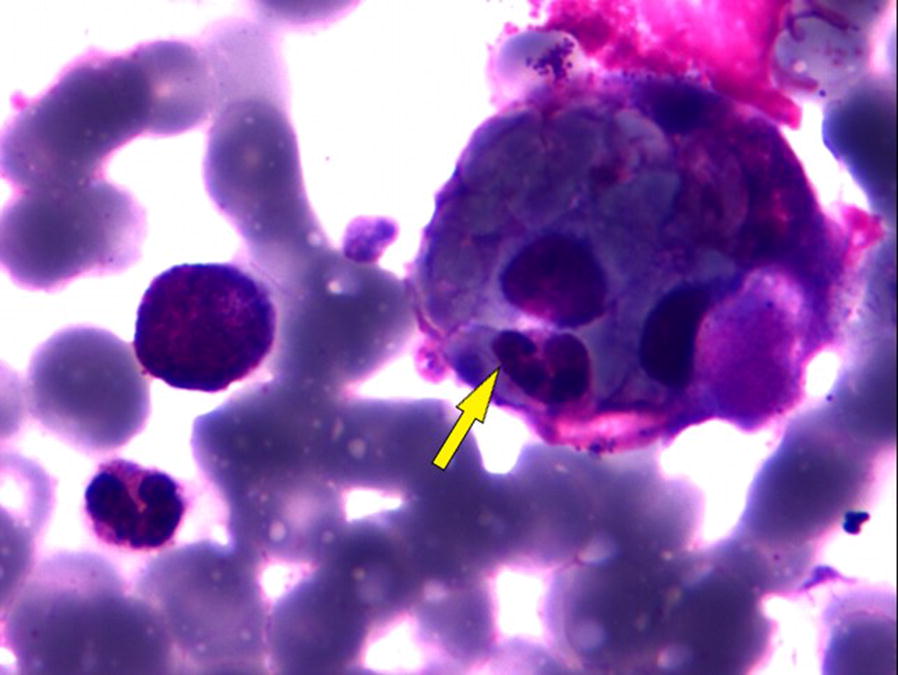
Wright Giemsa stain of the patient’s bone marrow aspirate with an arrow highlighting a macrophage phagocytizing red blood cells, neutrophils and platelets (original magnification, 1000)

On day 3 of hospitalization, the patient experienced shortness of breath and a very high fever (39.8 °C). An arterial blood gas test indicated pH of 7.55, P_O2_ of 57 mmHg, P_CO2_ of 36 mmHg and bicarbonate level of 31.6 mEq/L. Subsequently, the patient was intubated and connected to a mechanical ventilator. Due to the worsening clinical presentation, the ineffective treatment with broad-spectrum antimicrobials [cefoperazone-sulbactam (2000–1000 mg per day for 7 days) and azithromycin (250 mg per day for 2 days)], and continuing fever and cytopenia, HLH was suspected. A bone marrow aspirate was subsequently performed, which confirmed hemophagocytic activity (Fig. 1). Low natural killer (NK) cell activity (12.38%) and elevated serum soluble IL-2Rα (sCD25) levels (> 44,000 ng/L) also supported the diagnosis of HLH.

**Fig. 2:**
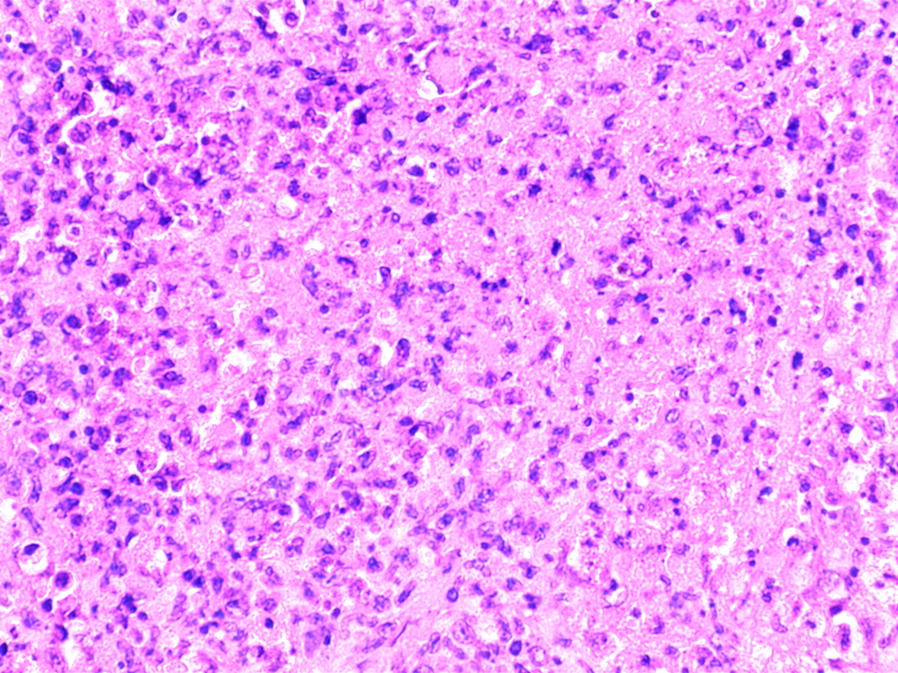
Inguinal lymph node with extensive geographic necrotizing granulomatous inflammation (hematoxylin–eosin, original magnification 600)

To identify the cause of HLH, a series of targeted examinations were performed. The patient had a negative T-SPOT test result. In addition, bacterial and fungal cultures were negative, and serology evaluations for Epstein–Barr virus, human herpesvirus-6, cytomegalovirus, influenza A virus, hepatitis B and C, human immunodeficiency virus and mycoplasma pneumoniae antibodies all yielded negative results. However, an inguinal lymph node biopsy was performed, and the histopathologic examination revealed necrotizing granulomatous inflammation (Fig. 2). The immunohistochemical stains of the lymph node revealed positive reactions to CD3, CD20, CD68, CD30, MPO, CD15 and Ki-67, which excluded the possibility of lymphoma. However, Warthin-Starry staining revealed negative results for the presence of organisms. Therefore, metagenomic next-generation sequencing (mNGS) analysis of the lymph node was performed. DNA of samples were extracted from lymph node homogenates with a TIANamp Micro DNA Kit (DP316, TIANGEN BIOTECH) according to the manufacturer’s recommendation. A total of 100 ng of the extracted DNA were subjected to processes of interruption, end repair, library construction, and sequencing. Sequencing were performed at BGISEQ-500 platform (Beijing Genomics Institute, Wuhan, China). A total of 44,544,787 single-end reads were generated from lymph node mNGS analysis. After filtering out the low-quality and human genome sequences (hg19), 32,259 microbial reads (0.07%) remained and were aligned to four Microbial Genome databases, consisting of 6350 bacteria, 1798 viruses, 1064 fungi and 234 parasites from the NCBI (https://ftp.ncbi.nlm.nih.gov/genomes/). A total of 7182 reads were discovered to be aligned to the *B. henselae* reference genome, with a genome coverage of 13.94% (280,820/2,014,762) (Additional file 1: Fig. S1). The other microbial sequences detected in the sample were mostly due to common laboratory contaminants or environmental microbes. Subsequently, communication was made with the family of the patient. The patient’s hometown is Susong in Anhui, a rural central region in China. Notably, the family reported that 4 days after the patient presented with the first allergic symptoms, the patient had been scratched on their right hand by a street cat.

Under the impression that the patient had an HLH, she was administered with intravenous immunoglobulin (500 mg/kg per day) for 5 days, followed by methylprednisolone (10 mg/kg per day). The patient also continued to receive minocycline (100 mg per day) and rifampin combination therapy to treat the *B.* *henselae* infection for a month. Subsequently, both the fever and skin rashes subsided. On the fifth day, the patient was successfully extubated and began to gradually regain consciousness; the liver function returned to normal levels and pancytopenia was reversed within 2 weeks. The patient was successfully discharged from the intensive care unit and remained in good condition after 2 months of follow-up.

To identify the genetic cause of HLH, whole exome sequencing (WES) was performed by a commercial service from BGI-Shenzhen (Shenzhen, China) as previously described [4]. In brief, whole blood was collected from the patient after obtaining written informed consent. DNA was extracted from blood and 50 ng of the high-quality DNA of the patient was used to prepare library and exome capture using MGIEasy Exome Capture V5 Universal Reagent Kit. High-throughput sequencing was then performed for each captured exome library on the BGISEQ-500 platform according to the manufacturer’s instructions. After variants called by BGISEQ-500 basecalling software, all clean reads were aligned to the human reference genome (GRCh37/HG19) using BurrowsWheeler Aligner (BWA V0.7.12). The genomic variations, including SNPs and InDels, were detected using the HaplotypeCaller of GATK (v3.7.0), then annotated using SNPEff, 1000 Genomes Project (http://www.1000genomes.org/), Exome Variant Server (EVS; http://evs.gs.washington.edu/EVS/), the Exome Aggregation Consortium (ExAC; http://exac.broadinstitute.org; release 0.3) and the Combined Annotation-Dependent Depletion tool. In total, more than 100,000 annotated variants were identified, demonstrating a hetero/homo ratio of 1.33; these variants were subsequently filtered based on their frequency, location, functional consequences, inheritance pattern and most importantly, clinical phenotype. The results revealed a heterozygous synonymous mutation (rs2303116; c.528C>T; p. Arg165Arg) in the STXBP2 gene, which was predicted to be deleterious by SIFT, Polyphen2 and PROVEAN. In addition, the patient was also discovered to have a splice donor variant in the IRF5 gene (rs2004640; NM_032643.4: c.-12 + 2G>T), which was previously reported to be associated with systemic lupus erythematosus, rheumatoid arthritis and secondary HLH (OMIM 607218). Using Sanger sequencing, these two identified mutations were confirmed in the proband (as a heterozygous mutation) and the patient’s healthy sister (as a heterozygous mutation).

## Discussion

In the present case report, the patient presented with typical symptoms of hemophagocytosis syndrome, including fever, splenomegaly, pancytopenia, decreased NK cell activity, hypofibrinogenemia, hemophagocytosis, hyperbilirubinemia, and increased levels of ferritin, sCD25 and lactate dehydrogenase. However, determining the cause of HLH remained difficult. In adults, HLH is often caused by various infectious agents (Epstein-Barr virus, cytomegalovirus, parainfluenza virus, *Mycobacterium tuberculosis* and *Mycoplasma pneumoniae*), autoimmune diseases (adult onset Still’s disease and systemic lupus erythematosus) or malignant conditions (lymphomas) [1]. Several medications, such as lamotrigine, can also promote HLH. It was suspected that the likely trigger for HLH in the patient was an allergic reaction to TCM that they had been prescribed; however, the medical history of the patient revealed that the rash associated with HLH began upon the allergic reaction to the drug subsiding, and the characteristics of these two rashes were different. Moreover, the patient had experienced several multi-localized specific drug allergic reactions previously, but they had never presented with secondary HLH before.

Both clinical and laboratory evaluations did not provide any evidence to indicate an infection initially. However, the lymph node biopsy provided an important clue for the etiological diagnosis, because the pathological findings prompted the consideration of the possibility of CDS. The pathogen causing CDS is *B. henselae,* which is difficult to culture and is insensitively detected by anti-*B. henselae* IgM-ELISAs. Recently, Parra et al. [5] developed a novel real-time PCR assay to detect *Bartonella* spp. with high sensitivity and specificity; however, a commercial PCR kit remains unavailable in numerous regions, including China. mNGS is an unbiased approach that can theoretically detect all pathogens in a clinical sample, and it is especially suitable for rare, novel and atypical etiologies of complicated infectious diseases. For example, Cheng et al. [6] used mNGS to detect the pathogens present in seven patients with culture-negative infective endocarditis; the results revealed that the pathogens, including *B. henselae*, in all the samples could be detected by mNGS. In our patient, mNGS also served an important role in the final determination of the etiology, especially since Warthin-Starry staining produced negative results for the presence of organisms. In general, histology is regarded as the gold standard in the diagnosis of *B. henselae* infection [7]. However, the results are often affected by several technical factors, such as possible sampling error, the quality of histopathology slides of biopsies and the subjective judgment of the tester [8]. Moreover, our patient received empirical antibiotics treatment before biopsy; although azithromycin for 2 days did not cure the patient, many of the bacteria in lymph nodes may have been killed off. Therefore, the bacteria were scantily and sparsely distributed that might be overlooked during Warthin-Starry interpretation. Fortunately, mNGS is less affected by prior antibiotic exposure, because it can detect trace amounts of nucleic acid. So, mNGS may be a useful adjunct in identification of the causative organisms in conventional test-negative infection.

The majority of the infections caused by *B. henselae* are mild, rarely running a severe course. To the best of our knowledge, fewer than 2 HLH cases associated with *B. henselae* infections have been reported to date, both of which occurred in immunocompromised patients [2, 3]. However, this patient denied suffering from any previous immunodeficiency disease or using immunosuppressive agents. Although allergic or adverse reactions caused by TCM are not considered to be the cause of HLH, allergic episodes to drugs may have a negative impact on the immune system; it is well established that both macrophages and T lymphocytes can be activated during allergic reactions [9, 10]. The pathophysiological mechanism of HLH has been found to be mainly due to the uncontrolled activation of cytotoxic T lymphocytes and macrophages, which subsequently induces the excessive production of cytokines [11]. Unfortunately, the relationship between the immune system disorder caused by the allergic reaction and the occurrence of HLH in this patient was not verified in the present study.

The pathogenesis of secondary HLH is not as understood as primary HLH. Although the likelihood of identifying a gene mutation is highest in younger patients, our genetic testing revealed two gene variants, STXBP2 and IRF5, in this adult patient with secondary HLH. STXBP2 reportedly serves an important role in the cytotoxic granule exocytosis of NK/T cells and its variants have been proven to be responsible for the pathogenesis of an HLH subtype, familial HLH type 5 [12]. A synonymous mutation (rs2303116; c.528C>T; p. Arg165Arg) in the STXBP2 gene, which does not alter the protein sequence, was detected in our patient, but it was predicted to be deleterious by gene function analysis. Moreover, this mutation has been reported to be associated with HLH in the Chinese Han population. Yang et al. [13] found that the rs2303116 CT/TT genotype (OR 3.900; 95% CI 1.537–9.899; P = 0.009) was an independent risk factor for HLH pathogenesis by using multivariate logistic regression analysis. IRF5 serves as a master transcription factor for the activation of genes encoding proinflammatory cytokines [14]. Polymorphisms in the IRF5 gene have been associated with the susceptibility to autoimmune diseases. Recently, Yanagimachi et al. [15] genotyped three IRF5 single-nucleotide polymorphisms using TaqMan assays in 82 patients with HLH and 188 control subjects; the statistical analysis revealed a significant association between the GT/TT genotype at rs2004640 with secondary HLH susceptibility. In addition, the prediction of the protein structure indicated that these mutations tended to cause partial defects in the protein function rather than the complete loss of the protein, and this partial loss of function may explain the later age of HLH onset in adults. Furthermore, it should be noted that the same genetic mutation was found in the patient’s sister; however, the sister had never exhibited any HLH clinical symptoms. These indicate quite clearly that the occurrence of the secondary HLH may be the result of the interaction between the infectious trigger and the genetic defects, while WES is an invaluable tool for genetic diagnosis in both primary and secondary HLH patients.

The main limitation of this current study was that we failed to conduct a genealogical analysis, because the patient’s parents died a few years ago, while her sister was willing to take a testing for susceptibility genes by conventional PCR but refused further WES. This makes it difficult to identified novel genes and mutations associated with HLH in this study. In addition, *B. henselae* infection was diagnosed by mNGS, but an elevation of anti-*B. henselae* by ELISA was not performed due to the lack of commercial kits.

In conclusion, HLH is a rare and highly morbid condition in adults, therefore identifying the etiology of HLH is crucial. The present case study emphasized the importance of mNGS in obtaining an etiological diagnosis, which suggested that screening for *B. henselae* should be considered in patients with HLH, especially those with a pet at home. In addition, the genetic defects were discovered to not only be present in primary HLH, but also in secondary HLH, even in the elderly.

## Supplementary information

**Additional file 1: Fig. S1.** Genome coverage of detected *B. hensela* sequences.

## Data Availability

The datasets for the current study are available from the corresponding author on reasonable request.

## Abbreviations

HLH: Hemophagocytic lymphohistiocytosis
CSD: Cause cat-scratch disease
TCM: Traditional Chinese medicine
NK: Natural killer
mNGS: Metagenomic next-generation sequencing
WES: Whole exome sequencing
