# Supplementary material for: Hemophagocytic lymphohistiocytosis is associated with Bartonella henselae infection in a patient with multiple susceptibility genes
Source: Ann Clin Microbiol Antimicrob. 2020 Jun 9;19:28. doi: 10.1186/s12941-020-00370-2 (PMC7281694; doi:10.1186/s12941-020-00370-2)
Supplement: Supplementary file 1 — Additional file 1: Fig. S1. Genome coverage of detected B. hensela sequences. [file 12941_2020_370_MOESM1_ESM.docx]

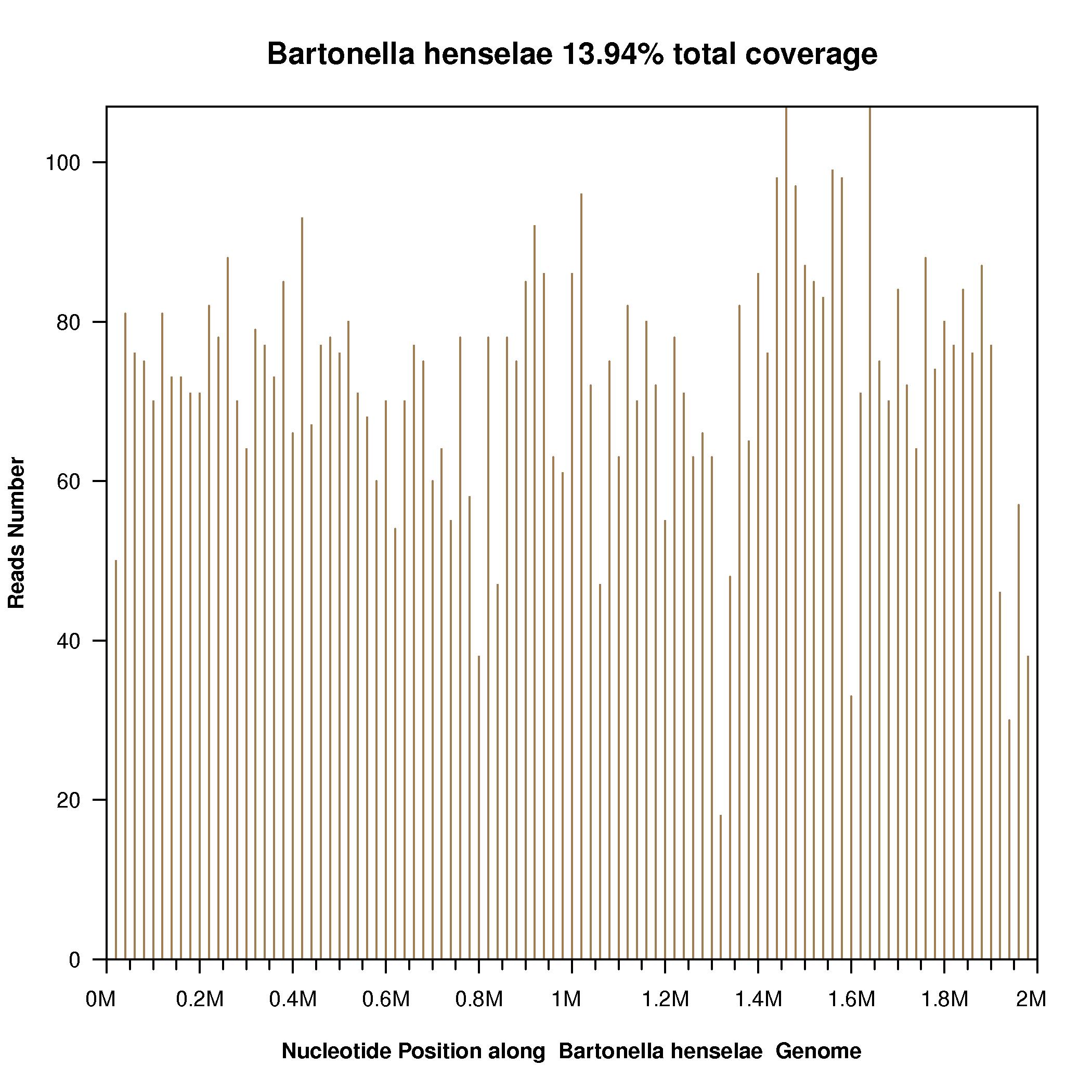


**Figure S1**. Genome coverage of detected *Bartonella henselae* sequences. A total of 44,544,787 single-end reads were generated from lymph node mNGS analysis. After filtering out the low-quality and human genome sequences (hg19), 32,259 microbial reads (0.07%) remained and were aligned to four Microbial Genome databases, consisting of 6,350 bacteria, 1,798 viruses, 1,064 fungi and 234 parasites from the NCBI (ftp://ftp.ncbi.nlm.nih.gov/genomes/). A total of 7,182 reads were discovered to be aligned to the *B. henselae* reference genome, with a genome coverage of 13.94% (280820/2014762). The other microbial sequences detected in the sample were mostly due to common laboratory contaminants or environmental microbes.
